# Left ventricular function assessment in Kawasaki disease by two-dimensional global longitudinal systolic strain with automated function imaging

**DOI:** 10.1186/s43044-024-00491-5

**Published:** 2024-05-19

**Authors:** Ehsan Aghaei Moghadam, Maryam Taraz, Aliakbar Zeinaloo, Mohammad Taghi Majnoon, Keyhan Sayadpour Zanjani, Mojtaba Gorgi

**Affiliations:** Cardiology Division, Pediatric Department, Children’s Medical Center (Pediatric Center of Excellence), Tehran University of Medical Sciences, No. 346, Keshavarz Blvd, Tehran, 14188 Iran

**Keywords:** Kawasaki disease, Automated function imaging, Children, Cardiac involvement

## Abstract

**Background:**

Kawasaki disease is an acute febrile vasculitis of childhood mainly affecting children under 4 years of age. In the acute stage of the disease, heart function decreases and gradually returns to normal after treatment. However, subendocardial involvement may persist, which cannot be assessed by M-mode echocardiography. Strain echocardiography is a recently developed technique to assess subendocardial involvement of myocardial deformation. We aimed to study the stratified strain of left ventricular function in a Kawasaki patient at least 6 months after the acute stage of the disease with special conditions for entering the study using two-dimensional speckle-tracking imaging. Between September 2020 and October 2022, 27 healthy children and 27 children with a history of Kawasaki disease more than 6 months ago were evaluated using two-dimensional global longitudinal peak systolic strain with automated function imaging technology.

**Results:**

The mean age of patients was 5.6 years. With M-mode echocardiography, ejection fraction of each group was in the normal range. Mean (± standard deviation) global longitudinal peak strain in four-chamber view of girls with Kawasaki disease was − 23.74 ± 2.77, and that in boys with Kawasaki disease was − 20.93 ± 2.06 (*P* value = 0.008). GLPS (global longitudinal peak strain) was compared as an overall average and as in a separate segment, which showed significant difference in two comparisons. In our study, a decrease in the function of some cardiac segments is reported. Global longitudinal peak strain in four-chamber view was significantly lower in boys. Comparing different segments, a difference in global left ventricular long-axis strain was found between the two groups. On the other hand, there was a major difference between the two groups in the basal inferolateral, basal anterolateral, and mid-inferolateral, which receives blood from Left Circumflex artery.

**Conclusion:**

Using stain echocardiography to detect continued subendocardial involvement in asymptomatic children with a history of Kawasaki disease for a better understanding of the condition, effective management and follow-up is recommended.

## Background

Kawasaki disease (KD) is an acute, self-limited medium vessel vasculitis, especially in boys younger than 5 years, that favors the coronary arteries [1]. In the past three decades, KD has become widespread, especially after COVID-19 pandemics, making it the leading cause of acquired heart disease in children. Small- and medium-sized arteries, particularly coronary, are the main targets. Although there is a great tendency for coronary assessment in KD, it should be kept in mind that other cardiac involvements such as myocarditis can have great impact on what we consider cardiac sequels. Some subclinical and also clinical myocardial involvements can cause permanent irreversible changes to the myocardium, and there should some assessments for detecting such a subtle change. Even histopathologic involvement in pericardium can also be a reason for future myocardial segmental dysfunctions. Aggressive methods like myocardial biopsy are not accepted methods to define the myocardial pathology and make an accurate diagnosis. So other noninvasive effective methods are accepted.

In this study, two-dimensional speckle-tracking imaging was used to assess LV longitudinal systolic strains in children with KD. Speckle-tracking imaging is a relatively newer and advanced technique for myocardial assessment as a global and regional unit. It is an independent technique that does not rely on the underlying ventricular geometry or morphology and the angle between myocardial movement. Echocardiographic evidence of decreased left ventricular (LV) systolic function is included as supporting evidence in the American Heart Association algorithm designed to improve the diagnosis of KD. The traditional method most commonly used to measure overall LV systolic function in children with KD is LV ejection fraction (LVEF). However, previous research has generally shown that LVEF is normal throughout the acute phase [2, 3]. Patients with coronary artery disease have previously been accurately identified using speckle-tracking echocardiography (STE) [4]. Furthermore, recent studies have shown aberrant strain in KD patients despite normal left ventricular ejection fraction [5].

STE can calculate strain globally and regionally. Compared to other methods such as tissue Doppler, regional strain is independent of the effects of translation and tethering. To facilitate clinical use, a strain calculation method called automated function imaging (AFI) was developed. AFI produces a parametric image of myocardial strain throughout the entire LV [6]. The purpose of this study is to examine LV function in patients with KD using two-dimensional global longitudinal peak strain with AFI technology.

For this reason, we decided to study the function of cardiac segments by the strain method in patients diagnosed with KD (based on the latest guidelines for the diagnosis of KD), who have been diagnosed with the disease for more than 6 months and who does not have confounding criteria, to see how the function of each heart segment changes after a month of illness with the removal of these effective factors, and to understand the relationship between each one and also find the parameters that influence cardiac function with strain. These criteria include: the absence of a history of KD or multisystem inflammatory syndrome in children (MISC) in the past, the absence of cardiac disease and myocarditis and glandular and metabolic diseases such as muscular dystrophy and chronic kidney disease and anemia, the absence of coronary artery ectasia or coronary artery aneurysm, the absence of a genetic syndrome such as Down or DiGeorge syndromes, and no cardiac medications use.

Based on our experience and global consensus, improving the level of echocardiographic assessment with STE may enable much earlier diagnosis of myocardial diseases such as KD and myocardial involvement caused by it, myocardial damage caused by chemotherapy drugs and similar diseases. This method deserves general attention worldwide [7, 8].

## Methods

In this case–control study, children with a history of KD and treated in the Pediatric Rheumatology Clinic between 2022 and 2023 were selected to evaluate left heart function considering the inclusion criteria. Inclusion criteria included age less than 18 years, a single history of KD (either complete or incomplete type), and at least 6 months have elapsed since disease onset.

Exclusion criteria included history of cardiac disease, rheumatic myocarditis, endocrine and metabolic diseases, muscular dystrophy, chronic kidney disease and anemia [9], history of Covid-19 or MISC in a child with KD (based on history and tests), history of coronary artery aneurysm or ectasia, the presence of genetic syndromes such as Down or DiGeorge, the use of cardiac medications, or parental unconsent. All patients met the diagnostic criteria for KD and were treated according to their condition.

The data including age, gender, weight and time interval from diagnosis to echocardiography, type of disease (complete/incomplete KD), type of treatment, clinical records and laboratory information were searched in the medical records. Two-dimensional global longitudinal peak strain was performed using AFI in 27 children with a history of KD and 27 healthy children matched for age and gender to the case group.

Echocardiography was performed in the standard two-, three-, and four-chamber views while the patient was in the lateral decubitus position, using the GE Vivid E9 echocardiography machine (General Electric Ultrasound, USA) and the 5-MHz probe. Digital loops were recorded on the machine's hard drive for offline and online analysis. The end of systole is considered the time of closure of the aortic valve in the apical long-axis view. The regions of interest are determined manually at the end of systole by marking the endocardial margin in the apical and parasternal views. Manual adjustments were made when automated tracking was not desirable. Segmental strain and average strain were automatically determined in all acoustic markers in each segment. The global longitudinal peak strain value was determined by averaging all segmental strain values from all three standard views, and EF was determined by the long-axis view and using the M-mode method. It should be noted that all echocardiographic examinations were performed by a single cardiologist.

Speckle-tracking echocardiography is a new technique that analyzes movement by tracking natural acoustic reflections within an ultrasound window. “Speckles” are stable patterns of 20–40 pixels that are automatically tracked throughout the cardiac cycle to follow myocardial motion and directly assess ventricular deformation in regions of interest [10]. Longitudinal left ventricular mechanics is the most sensitive component of left ventricular dynamics, and these components are most sensitive to the presence of myocardial disease [11]. The measurement of global longitudinal peak strain using speckle-tracking echocardiography is carried out from three ultrasound views. We captured three consecutive beats in each echocardiographic view using high-frame-rate harmonic imaging. Cardiac cycles were recorded as two-dimensional color video loops, and the acquired raw data were stored for offline analysis.

### Statistical analysis

Mean, standard deviation, frequency, and percentage were used to describe the data. Student T-test was used to compare quantitative variables between two groups, while Fisher's exact and Chi-square tests were used for parametric variables. Pearson's correlation coefficient was used to measure the correlation between variables. All analyses were performed by SPSS 25.0 statistical software (SPSS, Chicago, IL, USA). *P* value less than 0.05 was considered statistically significant.

## Results

Twenty-seven patients and 27 healthy children were entered into the study. Of the 27 participants, 11 (40.7%) were boys. The mean age of the patients and healthy children was 5.56 ± 2.2 and 5.73 ± 3.16 years, respectively. The mean weight of the patients and healthy children was 19.5 ± 6 and 17.4 ± 5.41 kg, respectively. No statistically significant difference between patients and control subjects in terms of gender, age or LVEF (M-mode) was found. Clinicodemographic data can be seen in Table 1. Table 2 includes data on symptoms, KD type, and treatment.

**Table 1:** Basic clinical and demographic data of patients and healthy children

Variable	Normal group (27)	Kawasaki disease group (27)
Male	11 (40.7%)	11 (40.7%)
Female	16 (59.3%)	16 (59.3%)
Age, mean (SD)	5.73 (3.16)	5.56 ± 2.28
Weight (kg), mean (SD)	17.4 (5.41)	19.51 ± 6.08
Heart rate, mean (SD)	101.93 (16.12)	100.81 (13.79)
EF, mean (SD)	63.40 (4.45)	65.26 (9.90)

SD, standard deviation; EF, ejection fraction

**Table 2:** Distribution of symptom and types of Kawasaki disease and treatment in patients

		N	%
Number of symptoms	1 symptom	13	(48.1%)
	2 symptoms	8	(29.6%)
	3 or more symptoms	6	(22.2%)
Disease type	Complete	19	(70.4%)
	Incomplete	8	(29.6%)
Treatment type	ASA	11	(40.7%)
	ASA + IVIG	16	(59.3%)

ASA, aspirin; IVIG, intravenous immune globulin.

In Table 3, the relationship between GLPS.LAX, GLPS.A4C, GLPS.A2C, and GLPS. Avg with gender, number of symptoms, disease type and treatment was evaluated. Mean GLPS.A4C in boys was significantly lower than girls. Mean GLPS.A4C of girls was − 23.74 ± 2.77, and that of boys was − 20.93 ± 2.06 (*P* value  = 0.008).

**Table 3:** Relationship between GLPS.LAX, GLPS.A4C, GLPS.A2C, and GLPS. Avg with clinical, demographical data*,* symptom and disease type. *P* value based on T-test

		GLPS.LAX	GLPS.A4C	GLPS.A2C	GLPS. Avg
Sex	Boy	− 22.03 ± 1.95	− 20.93 ± 2.06	− 23.7 ± 2.27	− 22.23 ± 1.14
	Girl	− 21.67 ± 7.13	− 23.74 ± 2.77	− 25.03 ± 3.42	− 22.37 ± 6.49
	*P* value	0.873	**0.008**	0.272	0.944
Number of symptoms	1 symptom	− 23.78 ± 3.78	− 22.41 ± 3.34	− 23.95 ± 2.43	− 23.41 ± 2.6
	2 symptoms	− 18.96 ± 8.2	− 23.75 ± 1.88	− 24.83 ± 2.84	− 20.34 ± 8.45
	3 symptoms and more	− 21.35 ± 2.87	− 21.47 ± 2.55	− 25.18 ± 4.56	− 22.57 ± 1.79
	*P* value	0.151	0.324	0.681	0.401
Disease type	complete	− 21.35 ± 6.28	− 22.81 ± 2.74	− 24.72 ± 3.26	− 22.02 ± 5.78
	Incomplete	− 22.91 ± 3.34	− 22.1 ± 3.2	− 23.94 ± 2.49	− 23.01 ± 2.31
	*P* value	0.516	0.566	0.553	0.644

ASA, aspirin; IVIG, intravenous immune globulin; BSA, body surface area; GLPS. LAX, global longitudinal peak strain (parasternal long axis); GLPS.A4C, global longitudinal peak strain (parasternal four-chamber); GLPS.A2C, global longitudinal peak strain (parasternal two-chamber); GLPS. Avg, global longitudinal peak strain (parasternal average). P value less than 0.05 was considered statistically significant

In Table 4, we depict cardiac parameters and regional strain in both genders. LVIDd and LVIDs in boys were significantly upper than girls. The values of segmental strain in mid-anteroseptal, mid-inferoseptal, apical-septal, apical-inferior, apical-lateral, apex were significantly lower in boys (all *P* value s < 0.05). Other parameters had no differences between two sexes.

**Table 4:** Regional strain in both genders. *P* value based on T-test

	Sex		*P*
	Boy	Girl	
FS percent	32.82 ± 7.78	36.69 ± 6.98	0.189
EF	61.91 ± 10.48	67.56 ± 9.1	0.148
LVIDs	22.55 ± 2.94	19.06 ± 3.13	**0.008**
LVIDd	33.82 ± 3.43	30.19 ± 4.43	**0.031**
Basal anterior	− 24 ± 5.4	− 23.5 ± 5.05	0.808
Basal anteroseptal	− 18.09 ± 3.21	− 19.63 ± 3.16	0.229
Basal inferoseptal	− 21.18 ± 2.6	− 21.56 ± 2.63	0.714
Basal inferior	− 20.18 ± 3.28	− 18.81 ± 5.38	0.460
Basal inferolateral	− 18 ± 4.8	− 19.31 ± 4.05	0.449
Basal anterolateral	− 22.09 ± 2.81	− 21.06 ± 4.81	0.530
Mid-anterior	− 26 ± 3.85	− 26.75 ± 5	0.679
Mid-anteroseptal	− 21.64 ± 1.96	− 24.44 ± 3.01	**0.012**
Mid-inferoseptal	− 22.91 ± 2.21	− 26.25 ± 3	**0.004**
Mid-inferior	− 19.82 ± 2.89	− 21.63 ± 7.29	0.444
Mid-inferolateral	− 19.45 ± 4.23	− 21.88 ± 4.79	0.189
Mid-anterolateral	− 25.09 ± 4.01	− 25.25 ± 4.88	0.930
Apical anterior	− 26.36 ± 3.07	− 28.88 ± 3.88	0.085
Apical septal	− 25.36 ± 3.5	− 29.81 ± 3.92	**0.006**
Apical inferior	− 24.45 ± 2.62	− 28.31 ± 4.45	**0.016**
Apical lateral	− 23.09 ± 3.7	− 28.12 ± 5.03	**0.009**
Apex	− 25 ± 2.19	− 29.06 ± 3.21	**0.001**

FS, fractional shortening; EF, ejection fraction; LVIDd and LVIDs, left ventricular internal diameter end diastole and end systole. P value less than 0.05 was considered statistically significant.

In Table 5, we present the mean regional strain between patients with complete and incomplete KD. The mean of segmental strain in basal inferoseptal and apical anterior had a statistically significant difference between the two groups.

**Table 5:** The mean cardiac parameters between patients with complete and incomplete Kawasaki diseases. *P* value s are based on T-test

	Disease type		*P* value
	Complete	Incomplete	
FS percent	35.79 ± 6.81	33.5 ± 9.02	0.475
EF	66.21 ± 8.69	63 ± 12.72	0.453
LVIDs	20.68 ± 3.64	20 ± 3.21	0.649
LVIDd	32.32 ± 4.61	30.13 ± 3.56	0.242
Basal anterior	− 24.42 ± 5.48	− 22 ± 3.82	0.268
Basal anteroseptal	− 19.32 ± 3.13	− 18.25 ± 3.49	0.442
Basal inferoseptal	− 20.79 ± 2.55	− 22.88 ± 2.1	**0.053**
Basal inferior	− 18.74 ± 5.01	− 20.88 ± 3.31	0.280
Basal inferolateral	− 18.05 ± 3.96	− 20.5 ± 4.93	0.185
Basal anterolateral	− 21.53 ± 4.49	− 21.38 ± 3.16	0.932
Mid-anterior	− 26.84 ± 4.81	− 25.5 ± 3.78	0.490
Mid-anteroseptal	− 23.58 ± 3.13	− 22.63 ± 2.5	0.453
Mid-inferoseptal	− 24.42 ± 3.27	− 26 ± 2.67	0.240
Mid-inferior	− 20.63 ± 6.31	− 21.5 ± 5.1	0.734
Mid-inferolateral	− 20.74 ± 4.01	− 21.25 ± 6.2	0.799
Mid-anterolateral	− 25.68 ± 4.77	− 24 ± 3.63	0.381
Apical anterior	− 28.84 ± 2.81	− 25.5 ± 4.72	**0.030**
Apical septal	− 28.53 ± 4.06	− 26.75 ± 4.89	0.337
Apical inferior	− 26.74 ± 3.87	− 26.75 ± 5.26	0.994
Apical lateral	− 26.63 ± 5.28	− 24.75 ± 4.77	0.394
Apex	− 27.47 ± 2.97	− 27.25 ± 4.65	0.881

FS, fractional shortening; EF, ejection fraction; LVIDd and LVIDs, left ventricular internal diameter end diastole and end systole. P value less than 0.05 was considered statistically significant

The correlation between GLPS.LAX, GLPS.A4C, GLPS.A2C, and GLPS. Avg variables with FS, E percent, LVIDs, and LVIDd was assessed. Based on analyses, only GLPS.A4C has a positive correlation with LVIDs. In other words, an increase of LVIDs increases the patient's GLPS.A4C (*P* value  = 0.038). There were no statistically significant correlations between other parameters.

In Table 6, the correlation between percent of FS, E percent, LVIDs, and LVIDd with segmental strain was investigated. LVIDd and LVIDs parameters had a significant positive correlation with segmental strain in mid-anteroseptal, mid-inferoseptal, apical anterior, apical inferior, and apex. For one-unit increase in LVIDs, the mentioned parameters increase by 0.47, 0.44, 0.40, 0.53, and 0.44 units, respectively. Also, for one-unit increase in LVIDd, the mentioned parameters increase by 0.40, 0.49, 0.36, 0.58, and 0.42 units, respectively.

**Table 6:** Correlation between percent FS, EF percent, LVIDs, and LVIDd variables with clinical parameters

		FS percent	EF percent	LVIDs	LVIDd
Basal anterior	Pearson correlation	− 0.264	− 0.280	0.305	0.182
	*P* value	0.183	0.156	0.122	0.363
Basal anteroseptal	Pearson correlation	− 0.189	− 0.190	0.245	0.178
	*P* value	0.346	0.343	0.218	0.375
Basal inferoseptal	Pearson correlation	0.127	0.148	0.100	0.264
	*P* value	0.528	0.463	0.619	0.184
Basal inferior	Pearson correlation	− 0.205	− 0.175	0.004	− 0.122
	*P* value	0.305	0.382	0.983	0.543
Basal inferolateral	Pearson correlation	− 0.041	− 0.006	− 0.046	− 0.065
	*P* value	0.837	0.977	0.820	0.747
Basal anterolateral	Pearson correlation	− 0.064	− 0.035	0.196	0.180
	*P* value	0.751	0.863	0.326	0.368
Mid-anterior	Pearson correlation	− 0.181	− 0.189	0.367	0.361
	*P* value	0.365	0.345	0.060	0.064
Mid-anteroseptal	Pearson correlation	− 0.287	− 0.289	0.470	0.401
	*P* value	0.147	0.144	**0.013**	**0.038**
Mid-inferoseptal	Pearson correlation	− 0.045	− 0.057	0.443	0.492
	P value	0.823	0.779	**0.021**	**0.009**
Mid-inferior	Pearson correlation	− 0.244	− 0.231	0.308	0.251
	P value	0.221	0.247	0.118	0.207
Mid-inferolateral	Pearson correlation	0.061	0.065	0.075	0.167
	P value	0.763	0.746	0.709	0.406
Mid-anterolateral	Pearson correlation	0.127	0.139	− 0.041	0.056
	P value	0.528	0.488	0.838	0.782
Apical anterior	Pearson correlation	− 0.228	− 0.258	0.405	0.369
	P value	0.252	0.193	**0.036**	**0.059**
Apical septal	Pearson correlation	− 0.136	− 0.140	0.288	0.290
	P value	0.499	0.487	0.144	0.143
Apical inferior	Pearson correlation	− 0.156	− 0.187	0.532	0.582
	P value	0.439	0.351	**0.004**	**0.001**
Apical lateral	Pearson correlation	− 0.067	− 0.067	0.156	0.165
	P value	0.742	0.739	0.438	0.410
Apex	Pearson correlation	− 0.203	− 0.220	0.444	0.426
	P value	0.309	0.269	**0.020**	**0.027**

FS, fractional shortening; EF, ejection fraction; LVIDd and LVIDs, left ventricular internal diameter end diastole and end systole. P value less than 0.05 was considered statistically significant

In the comparison of different segments, it was found that there is a major difference between the two groups in the basal inferolateral segment, which receives blood from left circumflex artery. However, other cardiac segments had similar conditions. On the other hand, there is no significant difference in global strain between the two groups of patients and healthy children (Tables 7 and 8).

**Table 7:** The correlation regional strain between case and control group

Variable	Kawasaki group		Normal group	
	Mean	SD	Mean	SD
**Left anterior descending**				
Basal anterior	− 23.70	5.10	− 23.80	5.61
Basal anteroseptal	− 19.00	3.21	− 18.60	3.09
Mid-anterior	− 26.44	4.50	− 26.27	4.11
Mid-anteroseptal	− 23.30	2.95	− 23.27	2.66
Apical anterior	− 27.85	3.73	− 26.20	2.70
Apical septal	− 28.00	4.31	− 24.60	4.10
Apex	− 27.41	3.46	− 24.87	3.14
**Right coronary artery**				
Basal inferoseptal	− 21.41	2.58	− 20.47	4.29
Basal inferior	− 19.37	4.62	− 18.07	8.19
Mid-inferoseptal	− 24.89	3.14	− 23.53	3.50
Mid-inferior	− 20.89	5.89	− 16.53	10.9
Apical inferior	− 26.74	4.22	− 24.60	3.85
**Left circumflex**				
Basal inferolateral	− **18.78**	4.33	− 21.47	2.50
Basal anterolateral	− 21.48	4.08	− 22.93	3.15
Mid-inferolateral	− 20.89	4.64	− 21.80	4.90
Mid-anterolateral	− 25.19	4.46	− 23.20	4.35
Apical lateral	− 26.07	5.12	− 22.40	2.91

SD, standard deviation. P value less than 0.05 was considered statistically significant

**Table 8:** Correlation global strain between case and control groups

Variable	Kawasaki group		Normal group	
	Mean	SD	Mean	SD
Global LV LAX	− 21.81	5.55	− 22.65	2.12
Global LV four-chamber	− 22.60	2.84	− 22.78	2.34
Global LV two-chamber	− 24.49	3.03	− 22.69	1.95
Average (Global LV LAX, Global LV four-chamber, Global LV two-chamber)	− 22.31	4.98	− 22.36	1.98

SD, standard deviation; LV, left ventricle.

## Discussion

Although there is a great tendency for coronary assessment in KD, it should be kept in mind that other cardiac involvements can have great impact on what we consider cardiac sequels. Some subclinical and also clinical myocardial involvements can cause permanent irreversible changes to the myocardium and there should some assessments for detecting such a subtle change. Even histopathologic involvement in pericardium can also be a reason for future myocardial segmental dysfunctions.

In this study, two-dimensional speckle-tracking imaging was used to assess LV longitudinal systolic strains in children with KD. Speckle-tracking imaging is a relatively newer and advanced technique for myocardial assessment as a global and regional unit. It is an independent technique that does not rely on the underlying ventricular geometry or morphology and the angle between myocardial movement. Generally left ventricular systolic function is preserved in many diseases, although myocardial involvement is present on echocardiographic measurements (EF, FS according to the M-mode method). As mentioned in previous studies in this field, the STE method can play an important role in the early diagnosis of cardiac involvement, especially in cases without coronary artery involvement where the disease progression is unusual. Stress echo is another method that can be used to check for abnormal myocardial contractility, but it is a complicated method that is not widely used.

According to our results, children with KD who were diagnosed more than 6 months ago and did not have any of the confounding factors in some segments compared to healthy children had a significant decrease in the functional range of the cardiac segments in each segment in longitudinal strain. The average strain also differed significantly with increasing age over 10 years. We found that the mean values of GLPS.A4C, LVIDd, LVIDs, and segmental strain in mid-anteroseptal, mid-inferoseptal, apical-septal, apical-inferior, apical-lateral, and apex were significantly lower in girls than in boys. Previous research mainly focused on LV systolic failure in the acute phase. It is well established that LV myocardial strain is measured by STE during the acute phase [12]. A more accurate marker of myocardial involvement in KD is longitudinal strain [13].

According to the Japanese Circulation Society, KD patients do not experience cardiovascular signs until 20 years after the disease occurrence [12]. Therefore, evaluating LV systolic function in asymptomatic children with a background of KD appears to be extremely important. For this reason, this study was conducted on patients for whom more than 6 months elapsed since their KD.

We observed that GLPS.A4C had a relationship with sex in KD and it was higher in boys. Taslakian et al. in population-based research in North American community found that of 121 children with KD, 61% were boys and younger KD children were at a higher risk than olders [15]. We found that in older KD patients, average GLPS had a significant difference with that of youngers. This finding shows that in patients with higher age, cardiac function is worse than in younger patients. This finding was different from Taslakian et al. study. Besides, we observed that males had higher cardiac complications than females. We found that LVIDs and LVIDd were higher in boys and mid-anteroseptal, mid-inferoseptal, apical-septal, apical-inferior, apical-lateral, and apex were significantly lower in boys than in girls.

In our study, we calculated the correlation between cardiac parameters. We found that LVIDs had a significant positive correlation with segmental strain in mid-anteroseptal, mid-inferoseptal, apical anterior and apical-inferior. In fact, for one-unit increase in LVIDs, the mentioned parameters increase by 0.47, 0.44, 0.40, 0.53, and 0.44 units, respectively. The correlation of LVIDd with segmental strain in mid-anteroseptal, mid-inferoseptal, and apical-inferior was observed as for one-unit increase in LVIDd, the mentioned parameters increased by 0.40, 0.49, 0.36, 0.58 and 0.42 units, respectively. These correlations were reported in children with KD for the first time as far as we know.

## Limitations

**Fig. 1:**
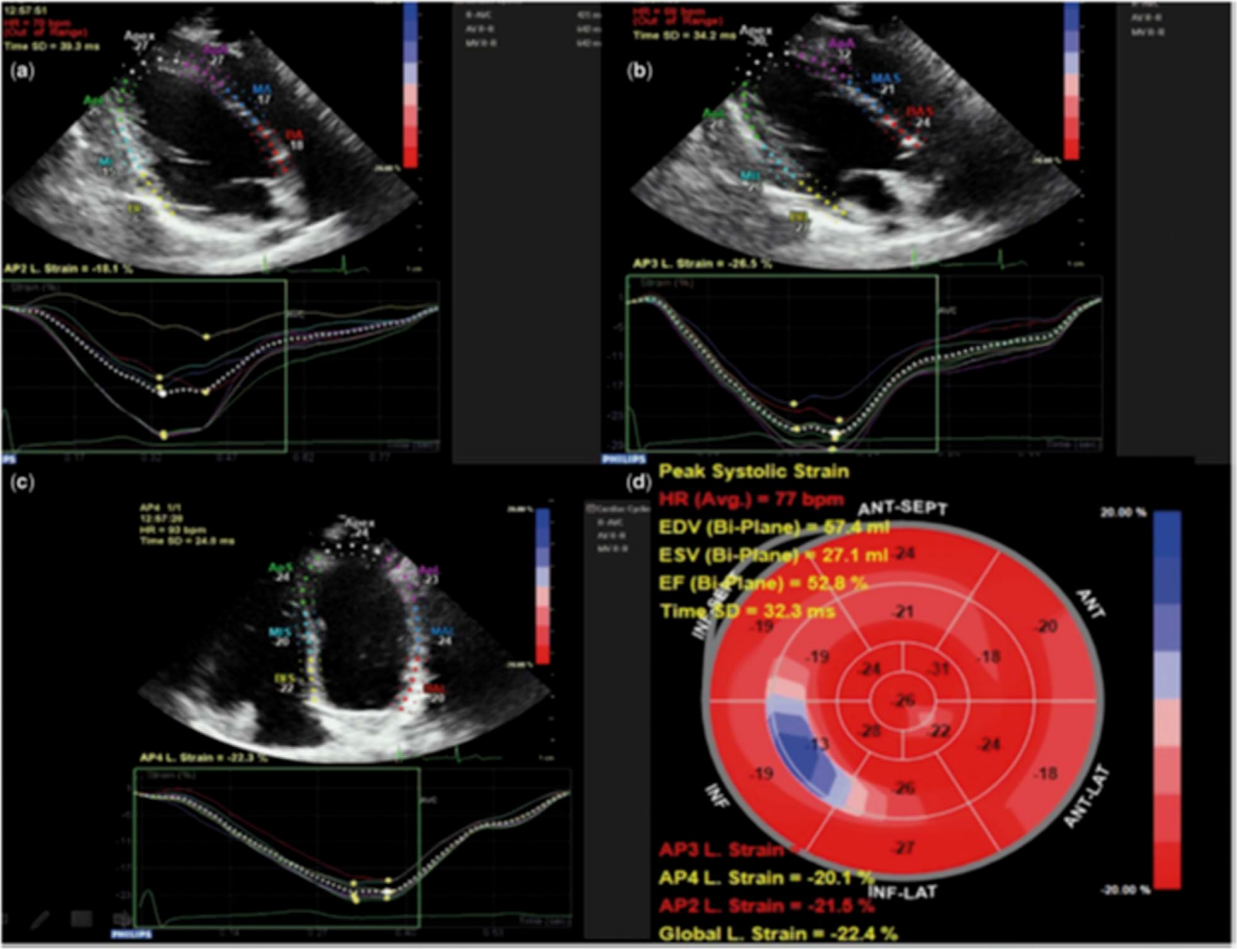
Automatically generated left ventricular deformation curves by the software (QLab). **a** Left ventricular longitudinal strain on the two-chamber view. **b** left ventricular longitudinal strain on the three-chamber view. **c** left ventricular longitudinal strain on the four-chamber view. **d** “Bulls Eye” schematic presentation of global strain measurements in left ventricular segments (11)

**Fig. 2:**
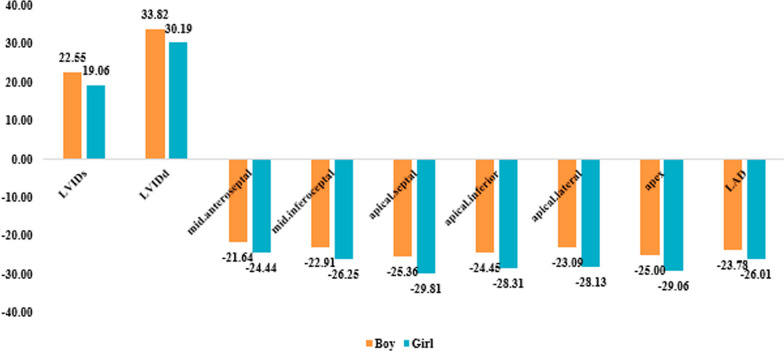
Regional strain in both genders

The number of patients was overshadowed by the reduction in patients referred to treatment centers due to COVID-19 pandemics. Besides, this study was single-center. In addition, due to the limited facilities, it was only possible to check the two-dimensional global longitudinal peak systolic strain. On the other hand, our conclusions would be strengthened if we could measure continuous longitudinal measurement data as well (Figs. 1, 2).

## Conclusions

Echocardiography uses STE to regularly check left ventricular function in patients with a history of KD is important regardless of coronary artery involvement. When examining cardiac function in KD, overall cardiac function improves over time, but the subendocardial region of the heart may not fully recover.

2D-STI could identify the degree of left ventricle systolic function in children with KD in different stage by evaluating myocardial stratification strain and among the related indexes of the strain, LV GLS had high accuracy as an indicator of early systolic dysfunction of LV myocardium in children in any stages of KD [16].

This study shows that despite the absence of outward symptoms in children with a history of KD, since there is a possibility of subendocardial involvement of the heart or re-engagement of the coronary arteries and the recurrence of the disease over time, follow-up examinations are extremely important. According to the physician's discretion, the smallest ischemic and heart function changes can be detected in the early stages by performing the global longitudinal peak strain method, which has a sensitivity of 95.8% and a specificity of 83.2% [16].

## Data Availability

The data that support the findings of this study are available from the corresponding author, Keyhan Sayadpour Zanjani, upon reasonable request.

## Abbreviations

ASA: Aspirin
IVIG: Intravenous immune globulin
BSA: Body surface area
GLPS.LAX: Global longitudinal peak strain (parasternal long axis)
GLPS.A4C: Global longitudinal peak strain (parasternal four-chamber)
GLPS.A2C: Global longitudinal peak strain (parasternal two-chamber)
GLPS. Avg: Global longitudinal peak strain (parasternal average)
LVIDd: LV end-diastolic diameter
LVIDs: LV end-systolic diameter.
KD: Kawasaki disease
LV: Left ventricle
LVEF: LV ejection fraction
STE: Speckle-tracking echocardiography
AFI: Automated function imaging
MISC: Multisystem inflammatory syndrome in children

## Declarations

### Competing of interests

Nothing declared.
